# Development of an XGBoost-based prediction model for wound recurrence risk in diabetic foot ulcer patients treated with antibiotic-loaded bone cement

**DOI:** 10.3389/fendo.2025.1610884

**Published:** 2025-07-29

**Authors:** Yi Zhang, Xingyu Sun, Cheng Cheng, Nianzong Hou, Shiliang Han, Xin Tang

**Affiliations:** ^1^ Department of Orthopedics, The First Affiliated Hospital of Dalian Medical University, Dalian, Liaoning, China; ^2^ Department of Hand and Foot Surgery, Zibo Central Hospital, Zibo, Shandong, China; ^3^ School of Mechanical and Electrical Engineering, Jining University, Jining, China; ^4^ Department of Medical Record Management, Zibo Central Hospital, Zibo, Shandong, China; ^5^ Center of Gallbladder Disease, Shanghai East Hospital, Institute of Gallstone Disease, School of Medicine, Tongji University, Shanghai, China

**Keywords:** XGBoost, decision curve analysis, feature selection, diabetic foot ulceration, antibiotic bone cement, diabetic foot, diabetes mellitus

## Abstract

**Background:**

This study aims to improve the surgical cure rate, develop interventions to reduce the incidence of postoperative nonunion or recurrence of diabetic foot wounds, and formulate an optimal prediction model to quantify the predictive risk value of antibiotic bone-cement failure in the treatment of diabetic foot.

**Methods:**

The training and test sets were created once the cases were collected. Based on feature correlation, feature importance, and feature weight, LASSO analysis, random forest, and the Pearson correlation coefficient approach were used to identify the features. Artificial neural network, support vector machine, and XGBoost prediction models were built according to the selected optimal features. The receiver operating characteristic curve, precision–recall (PR) curve, and decision curve analysis were utilized to validate the performance of the models and select the optimal prediction model. Lastly, an independent test set was created to assess and determine the best model’s capacity for generalization.

**Results:**

A comparative analysis revealed that the area under the curve (AUC) for the training set of the PRL-XGBoost prediction model was 0.85 and that for the test set was 0.71. This finding suggests that the model exhibits good predictive ability. Moreover, the PR-AUC value of the prediction model was 0.97, indicating that it demonstrates good resistance to overfitting. Additionally, the DCA curve showed that the PRL-XGBoost prediction model has significant application value and practicality. Therefore, PRL-XGBoost was found to be the most effective prediction model.

**Conclusions:**

The findings from this study prove that γ-glutamyl transpeptidase, lipoprotein A, peripheral vascular disease, peripheral neuropathy, and white blood cells are the key indices that affect the surgical outcome. These parameters determine the nutritional and immune status of the lower limb endings, leading to ulceration, infection, and nonunion of the diabetic foot. Hence, the PRL-XGBoost prediction model can be applied for the preoperative evaluation and screening of patients with diabetic foot treated with antibiotic bone cement, resulting in favorable clinical outcomes.

## 1 Introduction

Patients with diabetes mellitus (DM), a metabolic illness characterized by persistent hyperglycemia, may experience various problems. The prevalence of DM is increasing globally, assuming pandemic proportions in the 21^st^ century and emerging as a serious public health concern. Based on available data, 415 million individuals worldwide were projected to have DM in 2015. This figure is expected to rise steadily, reaching an estimated 642 million by 2040, with a 55% increase in the next 20 years (1). Diabetic foot syndrome refers to several typical pathological abnormalities, including infection, diabetic foot ulcer (DFU), and neuroarthropathy, which occur in the feet of patients with DM. DM leads to a major issue called diabetic foot (DF), which is strongly linked to peripheral vascular disease, neuropathy, and elevated foot mechanical pressure. The most prominent sign of DF disease is a foot ulcer, which has a very poor prognosis. DFU is a leading cause of illness and mortality, a significant public health issue, and a considerable burden to society. According to reports, foot care accounts for more than 20%–40% of diabetes-related medical expenses (2–4). DF adversely affects patients’ quality of life and their work performance (1). Patients with DF face immense physical and psychological effects as a result of their decreased ability to engage in regular activities.

Although many researchers have made significant progress in understanding the process and management of DF wound formation, DM and DF remain challenging issues that are likely to remain unresolved. Numerous factors influence the formation and incidence of DFUs, with peripheral neuropathy, peripheral artery disease, and local tissue infection being the primary factors that contribute to the pathogenesis of DFUs (5). All clinical practice recommendations for treating diabetic foot infection (DFI) wounds essentially follow the same steps, which include foot examination, antimicrobial therapy, debridement and dressing change, wound dressing, negative pressure wound therapy, revascularization, and patient and family education (4, 6, 7). However, DFI is highly difficult to treat, and the incision takes a long time to heal. Hence, hospital stays are often prolonged, and medical expenses increase. Antibiotic bone cement is a novel biological material that releases high concentrations of antibiotics locally, thereby reducing the risk of systemic toxicity and effectively preventing and treating infections. Interim studies have demonstrated that treating DFI wounds with antibiotic-loaded bone cement has yielded favorable therapeutic results. Researchers compared the use of antibacterial bone cement and vacuum sealing drainage in treating DF wounds using a case review research. They found that the former had a shorter healing period on average and a reduced infection rate on the 10^th^ day following surgery. Antibiotic bone cement can promote wound healing in patients with Wagner grade 3–4 DM by controlling infection and shortening the healing time (8).

Although antibacterial bone-cement surgery can help patients heal, it cannot predict risk factors, which leaves patients vulnerable to repeat procedures. Therefore, clinical prediction models have been widely utilized by researchers to identify risk variables and enhance surgical success rates. Clinical prediction models, often referred to as prognostic models, risk scores, or clinical prediction rules, are multivariate models used to determine the likelihood of a disease or its prognosis. Prognostic and diagnostic evaluations are included in the model. The prognostic model examines the possibility of complications, death, disability, and recurrence based on the current state of the disease at a given point in time (9). By building clinical prediction models, several researchers have identified risk factors for DF in patients with DM. To facilitate early diagnosis and screening, Wang (10) and Jing (11) developed a nomogram prediction model for risk variables associated with DF in individuals with type 2 diabetes. A predictive nomogram model was proposed by Peng (12) and Dai (13) to assess amputation risk in patients with DFUs. Furthermore, nanoformulations have received considerable research attention. Nanoformulations, such as antibiotic-loaded bone cement, have emerged as effective localized drug delivery systems for DFUs, ensuring sustained release and minimizing systemic side effects. Their integration in clinical practice enhances wound healing and reduces recurrence rates (14–17).

Although researchers have developed models to predict the risk of DF in patients with DM, recurrence risk prediction models constructed using machine learning algorithms for DFU after antibacterial bone-cement surgery have not been reported. By utilizing machine learning techniques to build predictive models, it is possible to conduct early screening, prevent diseases from developing, minimize serious complications, alleviate patient discomfort, and reduce societal and financial burdens. A predictive model can be designed to analyze the risk factors for recurrent amputation during the treatment of DF with antibiotic-loaded bone cement. Patients undergoing this treatment can be screened to prevent or reduce the probability of surgical failure recurrence and decrease the number of cases requiring amputation, thereby alleviating the pain and financial burden experienced by these patients. This approach would lessen the load on society; however, such research has not been conducted to date. Therefore, some case information was collected in this study.

**Figure 1:**
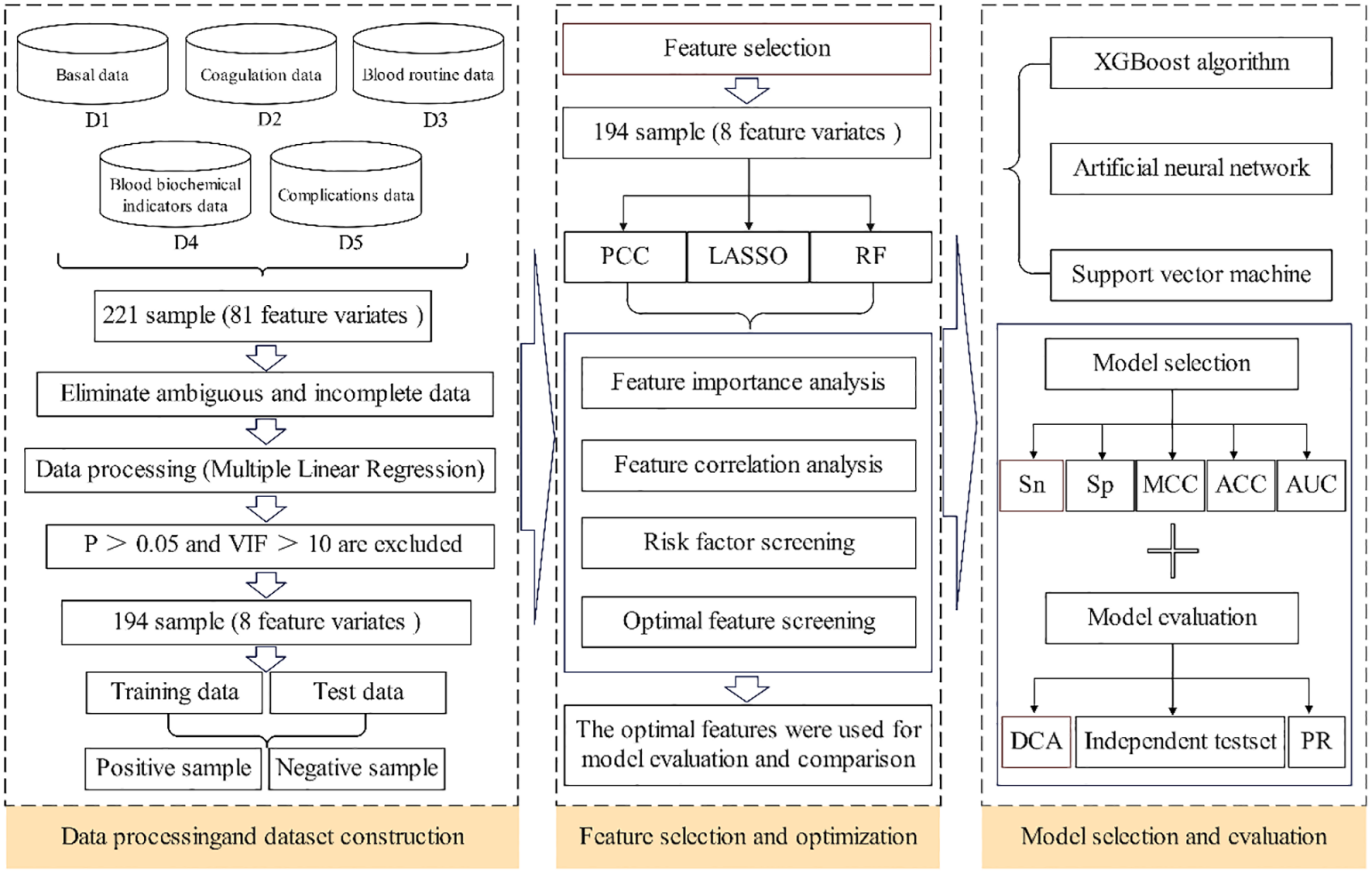
The overall framework of predict model.

Using a multi-feature machine learning algorithm, a prediction model was built to minimize pain and burden and perform early screening and prevention for patients with DF treated with antibacterial bone cement. This study aimed to develop a predictive model to estimate the risk of wound recurrence in patients with DF after surgical treatment with antibiotic bone cement. Ultimately, a PRL-XGBoost model was created using clinical data related to the risk of DF wound recurrence. As depicted in **Figure 1**, the approach was developed in three basic steps. First, analysis experiments were performed to create a dataset with high-quality clinical information. Analysis indexes of several patient clinical samples, including coagulation, blood lipid, blood glucose, electrolytes, liver function, and renal function, were included in the dataset. Using multiple linear regression (MLR) to identify vital features from the dataset, clinical datasets with P>0.05 and variance inflation factor (VIF)>10 were eliminated. The dataset was categorized into two parts: the training dataset and the test set. Second, optimal features were extracted from the vital features by merging multiple feature selection methods. Third, the risk of DF wound recurrence was predicted using three popular prognostic models containing the optimal features. Ultimately, the performance of the models was evaluated to determine the most suitable model.

## 2 Materials and methods

### 2.1 Dataset collection and preprocessing

To train and assess the prediction models, a comprehensive and carefully selected dataset is essential. In this study, patients diagnosed with type 2 diabetes and treated for DF at Zibo Central Hospital were enrolled. All patients underwent surgery using bone cement loaded with antibiotics. From 2020 to 2024, all relevant medical data were extracted from the hospital’s record system.

#### 2.1.1 Sample inclusion criteria

- Individuals with type 2 diabetes who met the specific criteria for diagnosis (fasting plasma glucose≥7.0 mmol/L, 2-h plasma glucose ≥11.1 mmol/L, or glycosylated hemoglobin ≥6.5%).
- Problems related to DF; The severity of DF was assessed according to Wagner grading, and the included patients were evaluated as Wagner grades 3–4.
- Verbal agreement from the patient.

#### 2.1.2 Sample exclusion criteria

Type 1 diabetes, gestational diabetes, thromboangiitis obliterans, tumors, and inadequate clinical data.

The 217 samples obtained from 5 categories—basal, coagulation, blood routine, blood biochemical indicator, and complications—were included in the sample clinical data. A total of 81 features (total of species) were gathered from the original sample, and the features of each of the five types (D1–D5) of samples differed as well. **Table 1** displays the initial data set.

**Table 1:** The sample of initial data sets.

Type	Category	Species	Sample size
D1	Basal data	12	217
D2	Coagulation data	6	198
D3	Blood routine data	21	211
D4	Blood biochemical indicators data	34	202
D5	Complications data	8	201

The noise (such as missing values and outliers) from the samples was removed with the MLR so as to eliminate sample feature vectors from the initial dataset. The VIF value had to be<10, and the significance (P value) had to be<0.05. Finally, 194 samples with eight features satisfied the P value and VIF value requirements. **Table 2** displays the VIFs and p-values for these eight attributes. The following criteria were applied when creating the 194 samples:

- There were 194 samples in total, including 132 samples assigned to the training set and 62 samples assigned to the test set. In order to maintain a balance between the training and test sets, the ratio was set to 2:1.
- Samples classified as positive or negative were separated based on the outcome of the operation. Successful operations were considered positive and vice versa.
- The independent test set was created by gathering 30 clinical patient data points in order to assess the over-fitting bias.
- In order to avoid any over-fitting in the prediction model, 10-fold validation was applied for the training and testing processes.

**Table 2:** The VIFs and p-values of the eight characteristics.

Independent variables	Unstandardized coefficients		Standardized coefficients	T	Sig.	VIF
	B	Std. Error	Beta			
Onset	0	0	0.211	2.354	0.02	2.087
WBC	0.042	0.019	0.341	2.18	0.031	6.361
BASO.P	-0.725	0.273	-0.486	-2.656	0.009	8.704
GGT	0.003	0.002	0.248	2.081	0.04	3.691
LP(a)	0	0	0.279	3.043	0.003	2.189
Drink	0.313	0.13	0.307	2.41	0.018	4.217
Vascular	-0.422	0.161	-0.42	-2.621	0.01	6.675
Nerve	0.317	0.155	0.314	2.041	0.044	6.145

### 2.2 Methods

#### 2.2.1 Feature extraction

An essential component of machine learning is feature extraction. Generally, the choice of various features has a huge impact on the outcomes of the predictions. In this study, most features associated with the prediction of the risk of DF wound recurrence were considered extensively. Three types of effective feature encoding methods were adopted: Pearson’s correlation coefficient (PCC) (18), Random Forest (RF) (19), and Least Absolute Shrinkage and Selection Operator (LASSO) (20).

Meanwhile, feature selection improves a model’s capacity for generalization by efficiently eliminating superfluous and irrelevant features. Effective feature selection was essential for developing strong machine-learning models and was applied to enhance these models’ predictive capabilities. Presently, a multitude of algorithms are employed to address feature-selection issues, which can be categorized into three groups based on the approaches used, such as filter, wrapper, and embedding algorithms. To improve the feature-selection efficiency, we proposed a powerful hybrid multi-feature (PRL) selection algorithm, which fused PCC, RF, and LASSO methods. The hybrid approach better integrated the feature selection and learning processes, whereas the embedded technique fully utilized the outcomes of the filter method. As a result, the fused method increased the accuracy of the final forecast while simultaneously swiftly eliminating any redundant and unnecessary features and selecting the best features.

#### 2.2.2 Pearson’s correlational coefficients

To examine the degree of linear correlation between data, statistician Karl Pearson created the correlation coefficient, a statistical measurement. The correlation coefficient can be defined in various ways based on the variations in the study objects, with PCC being the most commonly used definition. Generally, PCC finds the most important features, produces a correlation matrix, eliminates unnecessary data, and calculates the linear relationship between two continuous random variables. A perfect, but negative correlation is represented by a PCC of -1, and a complete nonlinear correlation between the two sets is represented by a PCC of +1. The PCC of a pair of variables, *x* and *y*, can be defined as Equation (1):


(1) $${\text{P}}_{xy}=\frac{\sum_{i}\left({\text{x}}_{\text{i}}-\bar{\text{x}}\right)\sum \left({\text{y}}_{\text{i}}-\bar{\text{y}}\right)}{\sqrt{\sum_{i}{\left({\text{x}}_{\text{i}}-\bar{\text{x}}\right)}^{2}}\sqrt{\sum_{i}{\left({\text{y}}_{\text{i}}-\bar{\text{y}}\right)}^{2}}}$$

where, $\bar{x}\;$ and $\;\bar{y}\;$ indicate the means of variables x and y, respectively.

#### 2.2.3 RF algorithm

The RF algorithm is an integrated machine-learning method (21) that builds several unrelated decision trees via a random resampling bootstrap approach and the node stochastic classification strategy and then votes to provide the final prediction outcomes. The values of the other features were left unchanged, the values of the data outside the characteristic variable’s bag were randomly jumbled, and the OOB error (*B_n_*) of the decision tree was recalculated after the OOB error (*Bo*) for each decision tree, after which each characteristic variable involved in the decision tree operation was determined (22). Next, the scrambled features’ variable importance measure (VIM) was determined. The variable importance measures *V(F_A_)* for each feature *F_A_* has a decision tree number of *m*, and their expression is given by Equation (2), where a greater VIM indicates a more significant feature:


(2) $$V\left(F_{A}\right)=\frac{1}{n}\sum_{m=1}^{N}\left(B_{n_{i}}^{F_{A}}-B_{o_{i}}^{F_{A}}\right)$$

Where, the OOB error of decision tree *m* is represented by $B_{n_{i}}^{F_{A}}$ when any characteristic *F_A_* value is not scrambled, and by $B_{o_{i}}^{F_{A}}$ when any characteristic *F_A_* value is scrambled.

#### 2.2.4 LASSO

The LASSO was initially developed by Robert Tibshirani in 1996 (20). It is a useful method for conducting the feature selection and regularization procedures. For this purpose, the approach penalizes the coefficients of regression variables, decreasing some of them to zero, using a shrinkage (regularization) procedure. During the feature selection step, the variables that remain non-zero after the shrinking process were selected to be included in the model. The goal of this approach was to decrease the forecast error. Supposing the data (*x_i_, y_i_*) with *i* = 1, 2… N, *y_i_* is the corresponding dependent variable, and the variables are independent of each other. When the regression coefficient vector is represented as *β=* (*β_1_,β_2_*_…_ *β_p_*) *^T^*, LASSO estimation is defined as Equation (3).


(3) $$\begin{array}{c} \arg\min\\ \;\beta \end{array}\left\{\sum_{i=1}^{N}{\left(y_{i}-\sum_{j=1}^{m}x_{ij},\beta_{j}\right)}^{2}+\lambda \sum_{j=1}^{m}\left|\beta_{j}\right|\right\}$$

Where, the parameter for the penalty, denoted by λ, is satisfied at λ ≥ 0.

### 2.3 Prediction model

#### 2.3.1 Artificial neural network

ANNs have been applied in a variety of domains, including complex nonlinear function mapping, image processing, and pattern recognition, to address classification and prediction issues (23). ANNs have also gained popularity in the last few years owing to their high prediction accuracy and the capacity for data learning. Equation (4) describes how a single artificial neuron functions, where node *j* is connected to node *i* by multiplying the output of the front neuron, *x_j_*, by the weight *ω_ji_*. There are n front nodes connected to node *i* in total. The bias *b_i_* of node I is added to the summation of the products of all inputs and weights. The output *y_i_* of node *i* is then obtained by transferring the final summation via an activation function f. In this study, the hyperbolic tangent function and the linear function were applied as the activation functions for the hidden layer and output layer, respectively


(4) $$y_{i}=f\left(\sum_{j=1}^{n}\omega_{ji}x_{j}+b_{i}\right)$$

#### 2.3.2 Support vector machines

Vapnik invented the SVM, which has been effectively used in numerous fields (24). Regression and classification are two applications of artificial intelligence that employ the SVM algorithm. To get beyond the “curse of dimensionality,” the SVM was employed in this study. Equations (5) and (6) can be used to define the SVM, respectively:


(5)  w,b,ξξ*min12‖w‖2+C∑i=1n(ξ+ξ*)

(6) $$s.t.\left\{\begin{array}{c} w\emptyset \left(x_{i}\right)+b-y_{i}\leq \epsilon +\xi^{*}\\ y_{i}-w\emptyset \left(x_{i}\right)+b\leq \epsilon +\xi^{*}\\ \xi +\xi^{*}\geq 0,i=1,\ldots \ldots n. \end{array}\right\}$$

Where, ξ and ξ* are slack variables, ϵ is an insensitive loss function, and *C* is the punishment parameter.

#### 2.3.3 Extreme gradient boosting algorithm

A novel boosting-based ensemble learning technique called XGBoost has proven useful for both regression and classification (25). The approach is essentially an ensemble method based on gradient-boosted trees, and it is a learning framework based on boosting tree models. The prediction’s outcome is the total of the scores that *K* trees project, as stated in Equation (7):


(7) $$\hat{y_{i}}=\sum_{K=1}^{K}f_{k}\left(x_{i}\right),f_{k}\in F$$

Where, *F* is the set of functions containing all gradient boosted trees, *x_i_* is the ith training sample, and *f_k_(x_i_)* is the score of the *k*th tree.

### 2.4 Performance measure

Four key parameters–sensitivity (Sn), specificity (Sp), accuracy (Acc), and Mathews correlation coefficient (MCC) were selected to assess the prediction performance of the various models (26, 27). In addition, the precision-recall (PR) curve (28), the area under the receiver operating characteristic (AUC), and the receiver operating characteristic (ROC) curve were considered. In addition, a dataset was immediately subjected to decision curve analysis (DCA) in order to determine the range of threshold probabilities, model values, advantage magnitude, and model applicability (29).

## 3 Results

### 3.1 Feature optimization using various algorithms

#### 3.1.1 Feature optimization based on Pearson coefficients

When evaluating the risk of failure after surgical treatment of DFus with antibiotic bone cement, the prediction outcome is influenced either directly or indirectly by various input variables. To identify the statistically significant input variables, a statistical analysis was initially conducted. A Pearson correlation analysis was performed to determine the correlation coefficients between each input feature. In the upper right corner, the correlation coefficient and significance level are displayed. The higher the correlation between the two variables, the greater the number of asterisks. The scatter distribution of the correlation between two indicators is presented in the lower left corner. The diagonal line in the line chart represents the positive correlation distribution or the frequency distribution of the index.

**Figure 2:**
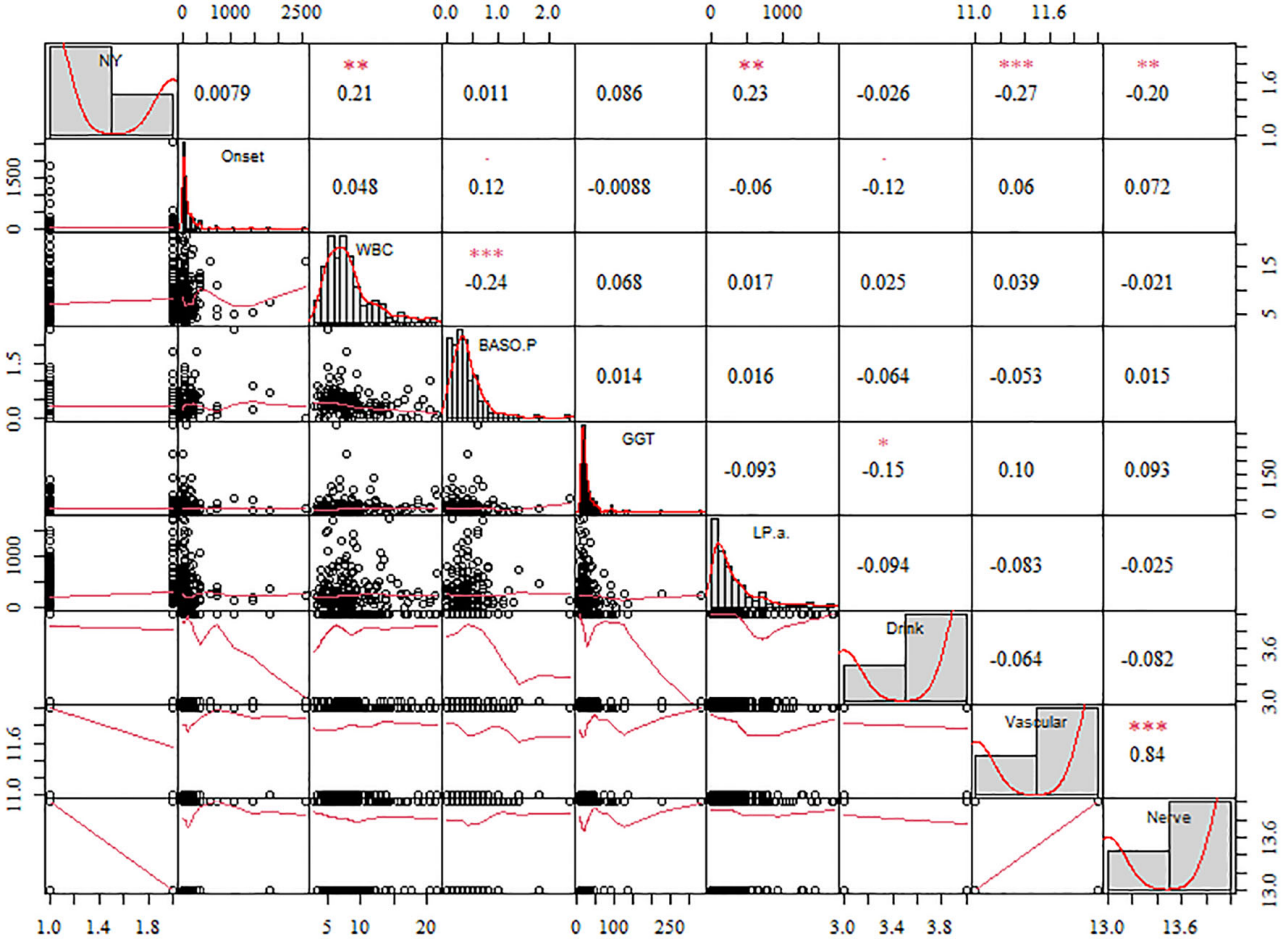
The correlation coefficient of the different features with the diabetic foot wound recurrence risk factors. The significance level are shown in the upper. The more asterisks, the stronger the correlation between the two variables.

**Figure 2** shows that lipoprotein A (LPA), white blood cell (WBC) counts, and γ-glutamyl transpeptidase (GGT) were positively correlated with factors related to the success of the surgery (NY), whereas Vascular and Nerve were negatively correlated. A comparison of the features revealed that LPA (0.23, P< 0.05) and WBC count (0.21, P< 0.05) exhibited a high positive correlation coefficient with the risk factors of DF wound recurrence. In contrast, Vascular (−0.27, P< 0.01) and Nerve (−0.20, P< 0.01) showed a high negative correlation. Furthermore, WBC and BASO.P exhibited a significant negative association (−0.24, P< 0.01), whereas Vascular and Nerve displayed a significant positive correlation (0.84, P< 0.01). To improve the prediction outcomes of factors related to surgical success, additional scoring weight methods must be employed for feature optimization.

#### 3.1.2 Feature importance from RF models

**Figure 3:**
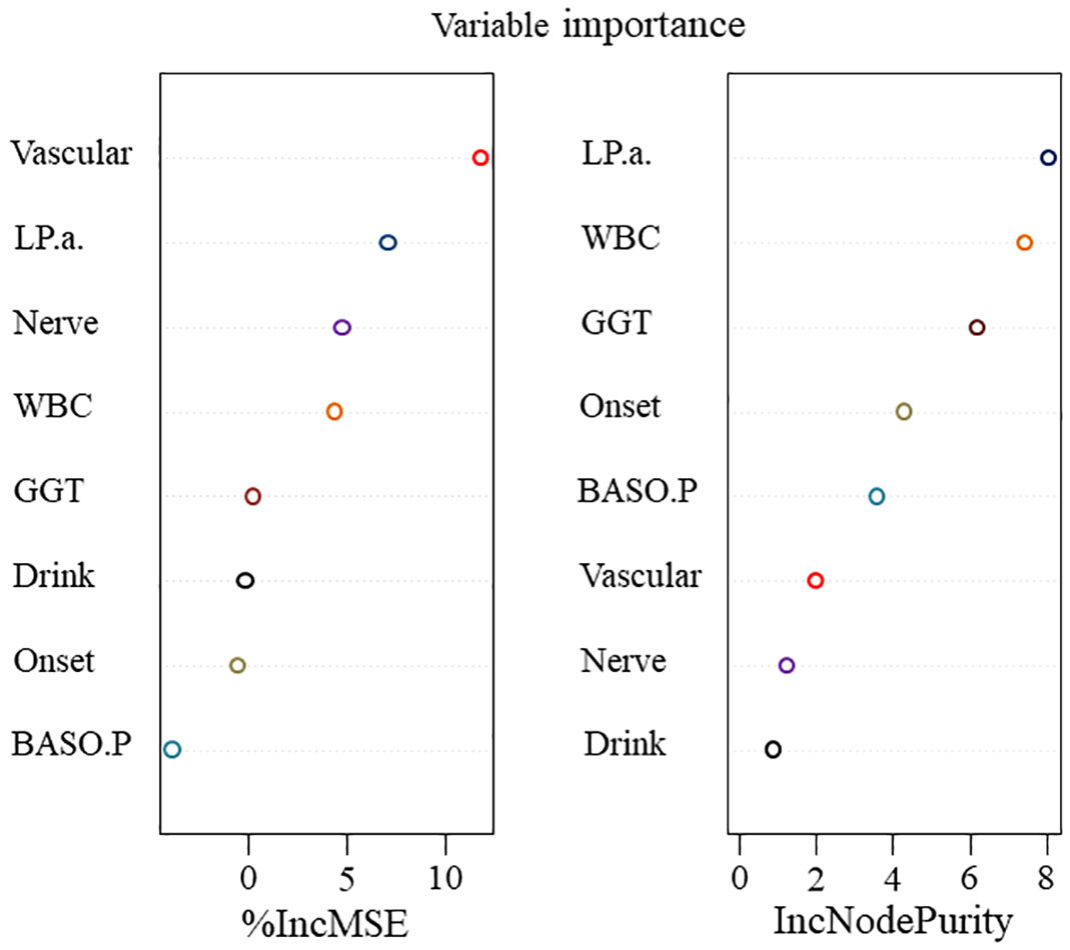
The importance of features in RF concerning information regarding features with factors related to the success of surgery.

Extracting a feature importance rank from tree-based models, such as RF, is another common feature selection technique. RF, a type of data mining model, exhibits a high degree of prediction accuracy and is often applied in data regression analysis, prediction, and classification tasks. Not all features considerably increase the regression accuracy in data regression analysis. Certain features are not readily apparent, which could lead to random noise in the regression and a significant model error. Hence, traits that contribute little should be eliminated. The key ranking results for RF attributes based on IncMSE and IncNodePurity (30) are illustrated in **Figure 3**. The greater the value of IncMSE, which represents the feature’s contribution to the target variable’s prediction accuracy, the more significant the feature is. IncNodePurity shows an increase in a node’s purity. A node with a higher purity level has fewer contaminants overall (i.e., a smaller Gini coefficient). To examine the relative importance of variables, IncNodePurity is also quantified by the sum of squares of residuals, which indicates how each feature affects the heterogeneity of observed values at each node of the classification tree. The variable’s significance increases with increasing value. The rankings of “%IncMSE” and “IncNodePurity” showed certain discrepancies, and the former was chosen as the index to assess the relevance of the predictive variables, signifying that the %IncMSE importance ranking results received greater weight. The features are significant indications of predictive features, as inferred from the considerable influences of Vascular, LPA, Nerve, WBC, and GGT on the predictive features.

#### 3.1.3 Feature optimization based on LASSO

**Figure 4:**
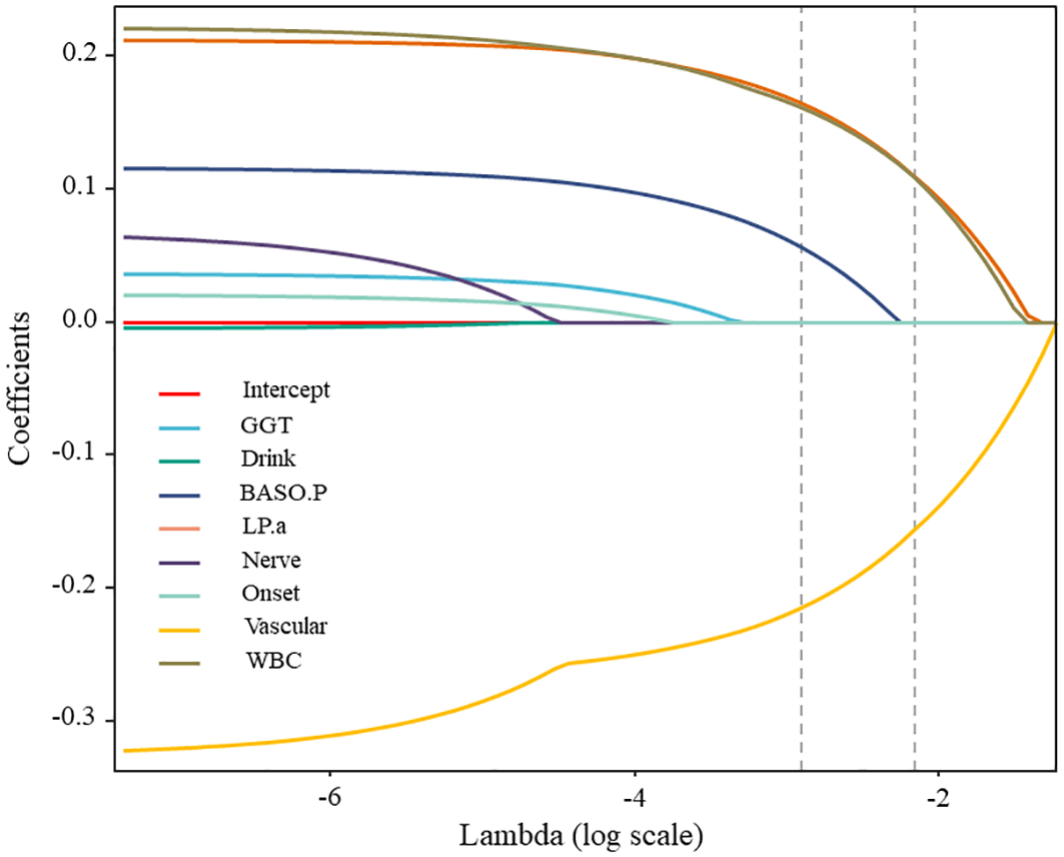
The variable selection procedure.

The LASSO method was utilized to eliminate pointless variables before selecting the ones to be included in the model. The results of variable selection are shown in **Figure 4**. As illustrated in the figure, LASSO compresses the regression coefficients of independent variables to establish a penalty function, thereby compressing unimportant variables to 0. The variables listed above the intercept are positively correlated with the DF wound recurrence risk factors. In contrast, those listed below are negatively correlated with factors related to the success of the surgery. The final compressed positive correlation factors were LPA, WBC, GGT, and Nerve, whereas the negative correlation variable was Vascular.

#### 3.1.4 Results of feature selection

Multiple linked features are often present in actual data, which makes the model unstable and prone to noise. The model’s ability to predict outcomes may be hindered by small changes in the data that significantly alter its output. In this study, the Filter and Embedded approaches were used to select the best characteristics for achieving the most accurate predicted outcomes. **Table 3** lists the outcomes of various techniques and demonstrates a strong correlation between factors related to surgical success and Vascular, LPA, Nerve, WBC, and GGT. Ultimately, five features were identified as optimal for predicting the likelihood of DF wound recurrence factors related to surgical success.

**Table 3:** The result of feature selection.

Method	Algorithm	The results of feature ranking	Optimal feature
Filter method	PCC	Vascular, LP.a, WBC, Nerve, GGT, Drink, BASO.P, Onset	Vascular, LP.a, Nerve, WBC, GGT
Embedded method	RF	Vascular, LP.a, Nerve, WBC, GGT, Drink, Onset, BASO.P	
	LASSO	Vascular, LP.a, WBC, Nerve, GGT, Onset, BASO.P, Drink	

### 3.2 Performance assessment with different prediction models

**Figure 5:**
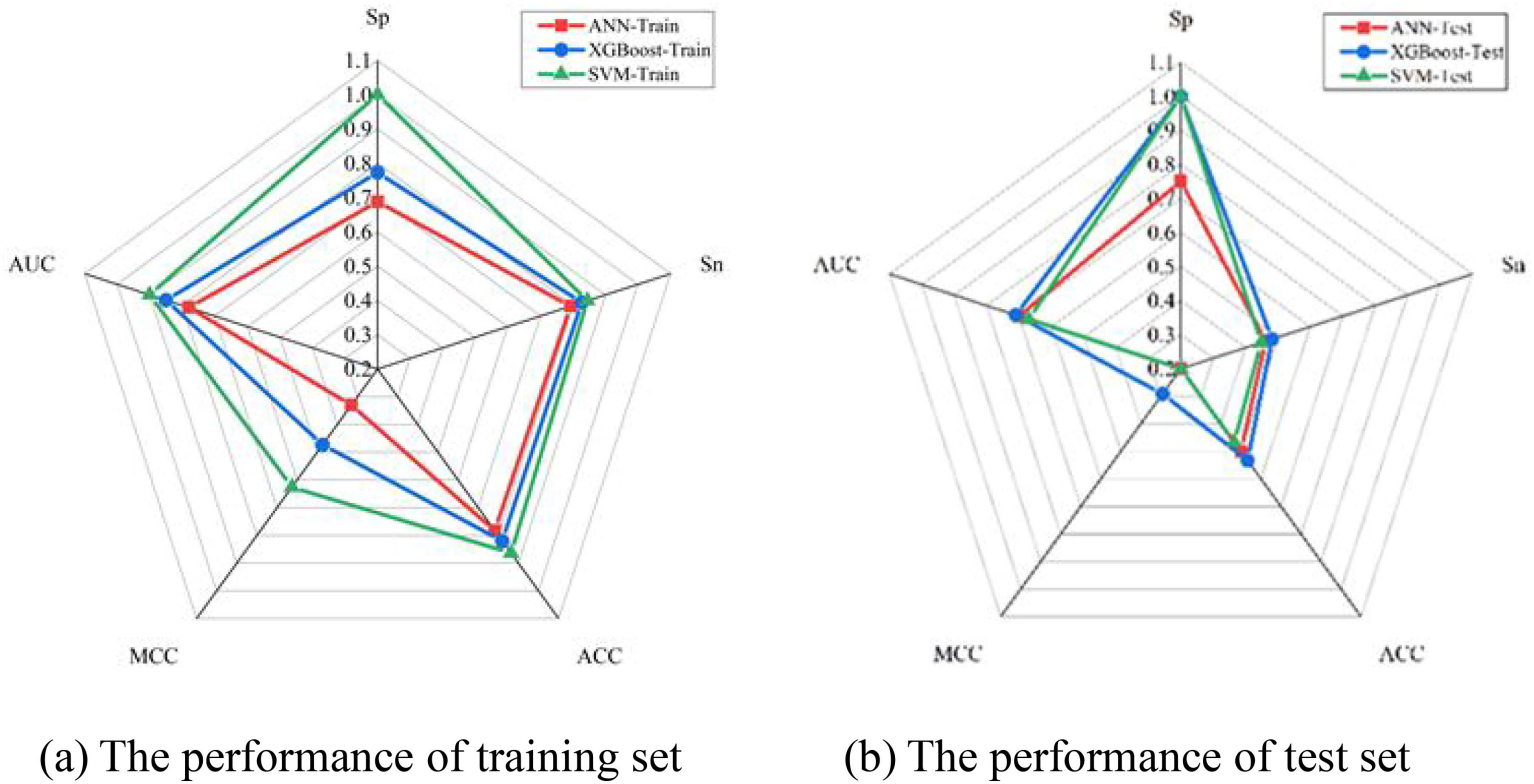
The graph shows the performance analysis of training and test set.

The performance of various production models was investigated based on five ideal features. Model performance was examined by constructing three prediction models: SVM, ANN, and XGBoost. **Figure 5** illustrates how the Sn, Sp, Acc, and MCC of the three models attained the desired performance on the training and test sets. As presented in **Figure 5a**, the SVM model with optimal features achieved the best outcome for the training sets. With a Sp of 1, Sn of 0.85, Acc of 0.86, and MCC of 0.63, the best results were obtained. As illustrated in **Figure 5b**, the test sets’ ideal outcome was achieved by the XGBoost model with optimal features. A Sn of 0.46, Acc of 0.5, and MCC of 0.16 represented the best results. By training the training set, the model modifies its parameters to perform increasingly better on the training set. The more extensive and vaster the training set, the more robust the model’s comprehension and data generalization capabilities. The test set is used to evaluate the model’s performance. To augment the generalization performance, optimal model parameters can be selected, and the model structure can be modified using test set–based performance evaluation. The XGBoost model was tested with optimal features. Consequently, emphasis was placed on the outcomes of the test set. **Figure 5b** and **Table 4** show that the XGBoost model achieved a superior trade-off between specificity and sensitivity.

**Table 4:** Comparison the performance of different model.

Method	Training/Test set	Training/Test set	Training/Test set	Training/Test set	Training/Test set
	Sp	Sn	ACC	MCC	AUC
ANN	0.69/0.75	0.79/0.46	0.78/0.5	0.33/0.15	0.78/0.7
SVM	1.0/1.0	0.85/0.45	0.86/0.47	0.63/0.16	0.9/0.67
XGBoost	0.77/0.75	0.82/0.46	0.82/0.5	0.47/0.16	0.85/0.71

**Figure 6:**
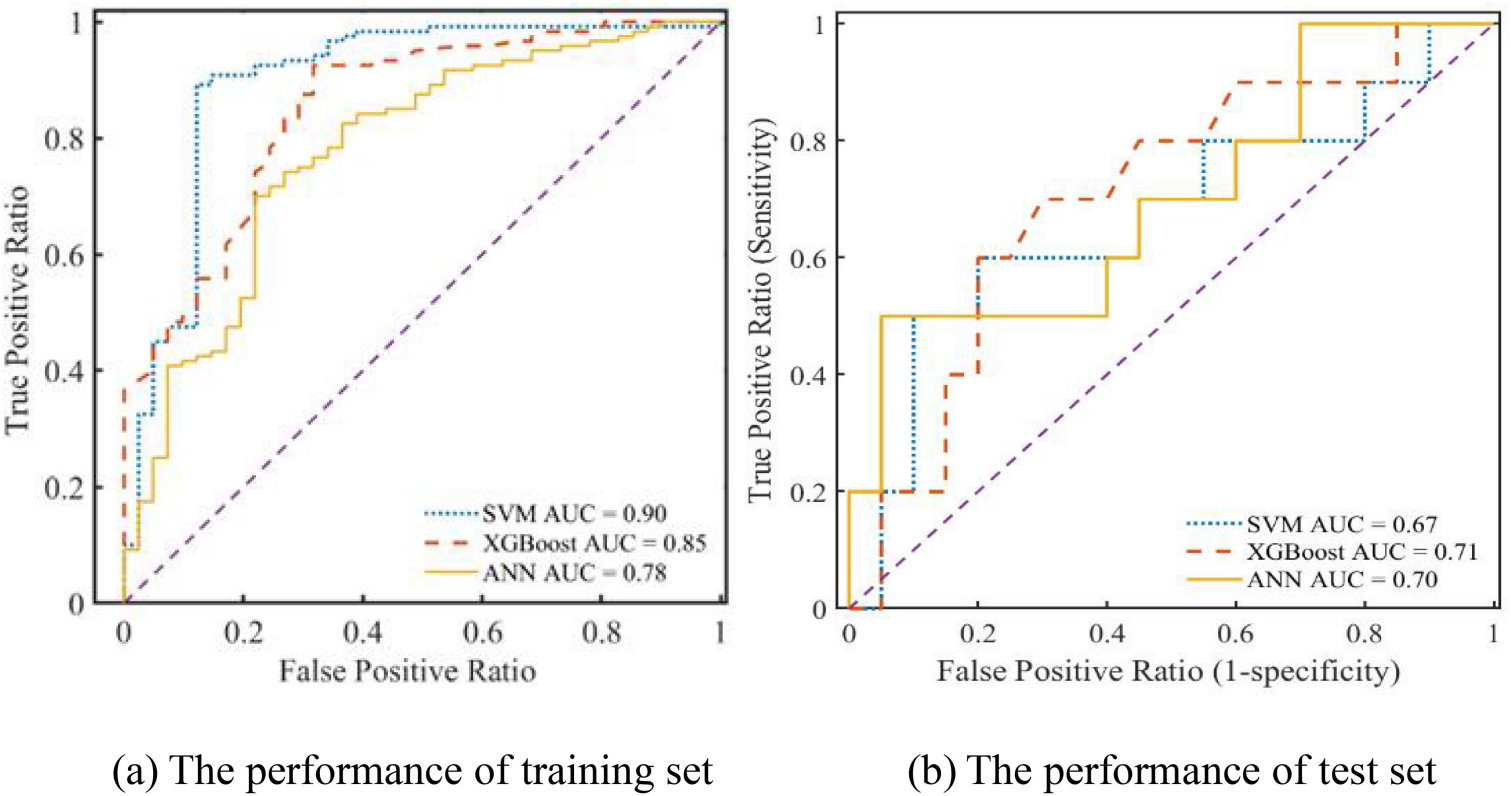
The ROC curve of different prediction models.

Furthermore, the prediction performance of the SVM, ANN, and XGBoost models was assessed using ROC curves and DCA. Upon comparing the outcomes from **Figure 6a**, it is evident that the SVM, ANN, and XGBoost AUC values of the models were 0.9, 0.78, and 0.85, respectively, for their training sets of ROC curves. On the contrary, the test set of ROC curves for the XGBoost model achieved the maximum value of 0.71, as shown in **Figure 6b**. Therefore, regarding prediction performance, the XGBoost model outperformed others owing to its increased stability and robustness.

**Figure 7:**
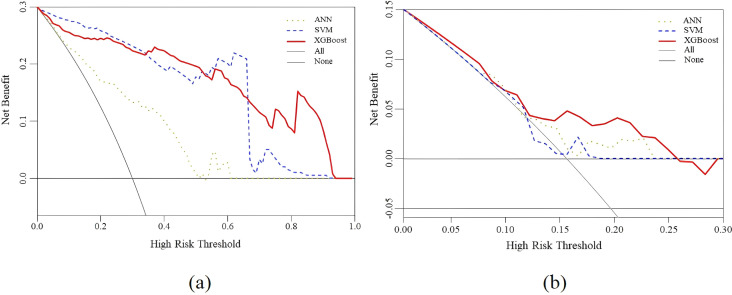
Decision curve analysis for the XGBoost, SVM and ANN prediction models. **(a)** The performance of training set **(b)** The performance of test set.

Finally, DCA curves were plotted to calculate the range of threshold probabilities, including whether a model is valuable, the magnitude of benefits it offers, and the best among several models. Typically, models with larger net worth returns are more useful in practice. In **Figure 7**, the *x*-coordinate is the threshold probability, and the *y*-coordinate is the model’s net benefit. The horizontal line and the gray line represent the two extremes, with the horizontal line representing zero intervention and a net benefit of 0. The gray line indicates the intervention for all forecasts. As inferred from **Figure 7A**, none of the three models exhibited a practical value beyond a threshold probability of 0.93 because the values of all three models’ training sets were 0. In addition, **Figure 7B** shows that none of the three models displayed a practical value beyond a threshold probability of 0.26 because the values of all three models’ test sets were 0. Overall, the net benefit of the XGBoost model was greater than 0 within the threshold probability range of 0–1, which increased the net benefit compared with that of other models, both with and without intervention. The test and training sets exhibited net benefits of 0.15 and 0.3, respectively, implying that XGBoost has good application value and practicality. Therefore, this model was selected as the appropriate predictor for constructing the optimal prediction model.

### 3.3 PR curve analysis using various prediction models

**Figure 8:**
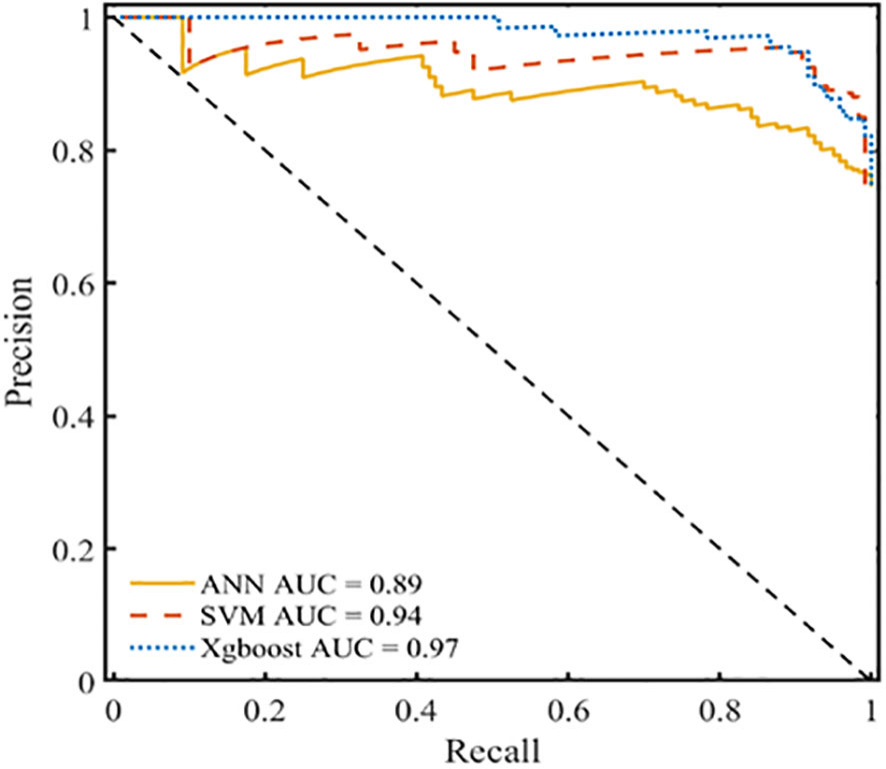
PR curve analysis for the XGBoost, SVM and ANN prediction models.

The ROC curve offers significant benefits as it can be utilized to evaluate classification difficulties with unevenly distributed samples because it is insensitive to variations in the ratio of positive to negative samples. The PR curve usually yields more useful information when samples are highly imbalanced. Recall and precision are represented on the X- and Y-axes, respectively, in the PR curve. The area under the curve (AUC) represents the overall performance of the model, and each point indicates the precision rate and recall rate below a specific threshold. The PR curve is used to assess the reliability of the developed model and confirm the effect of data imbalance on the prediction outcomes of various models. **Figure 8** shows that the areas under the PR curve (PR-AUCs) of ANN, SVM, and XGBoost models were 0.89, 0.98, and 0.97, respectively. The sample has no discernible effect on the model’s prediction outcomes, which further supports the idea that the XGBoost prediction model developed in this work exhibits excellent anti-data imbalance properties and can be applied to develop clinical predictions.

## 4 Clinical application of the model and discussion

### 4.1 Clinical application of the model

**Figure 9:**
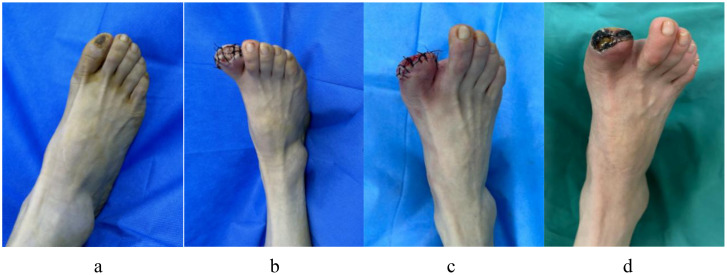
A 54-year-old woman with diabetic ulcers in her right great toe underwent an antibiotic bone cement procedure, but after a month the wound that was sutured after antibiotic bone cement be taken out did not heal and reinfects. **(a)** Preoperative ulcerations of the great toe of the right foot; **(b)** The right great toe was treated with debridement and amputation, then implanted with antibiotic bone cement; **(c)** Three weeks later, the antibiotic cement was removed, the wound was fresh, which was sutured directly; **(d)** One month later the wound did not heal, with necrosis and infection around skin margin of the wound.

Patients with DF are currently being treated at our facility using antimicrobial bone cement. Although the outcomes have been successful in some patients, many continue to experience nonhealing wounds or relapses that require reoperation. This observation is depicted in **Figure 9a**. A 54-year-old woman with a 15-year history of DM presented with diabetic ulcers in her right great toe as a result of nonunion following trauma. In the internal medicine department, the patient received treatment that included blood glucose management and dressing changes; however, the outcomes were not favorable. Right lower limb arterial embolism, inadequate blood glucose control, and increased white blood cell count were identified during preoperative evaluation. Recanalization could not be achieved via vascular surgery as the patient exhibited significant peripheral vascular embolism. After debridement, amputation of the necrotic portion of the great toe was planned, followed by antimicrobial bone cement, which was to be removed after 3 weeks, as seen in **Figure 9b, c**. Although the wound was new and sutured directly, the blood supply to the skin was weak. After a month, the sutured skin became necrotic and again infected, necessitating another amputation, as depicted in **Figure 9d**. To minimize the likelihood of surgical failure and alleviate patient suffering, data were collected from these patients to develop a clinical prediction model that identifies risk factors. The five most significant risk variables were GGT level, LPA level, peripheral vascular disease, peripheral neuropathy, and WBC count. The most successful prediction model, XGBoost, is currently utilized for patient selection and preoperative assessment, enhancing the probability of surgical success.

**Figure 10:**
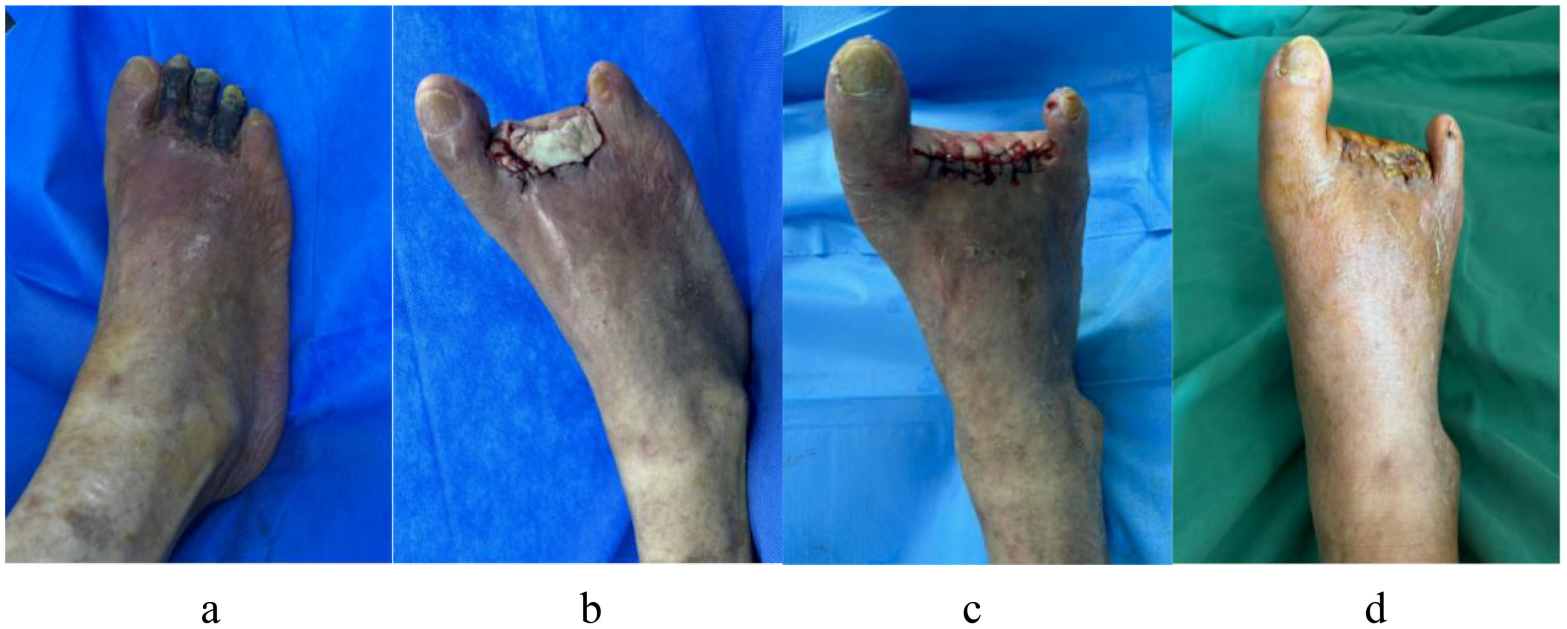
A 74-year-old man with a 24-year history of diabetes has diabetic gangrene in his right foot’s second to fourth toes, who accept resection of the necrotic toe and implantation of antibiotic bone cement, in the end the wound healed well. **(a)** Dry gangrene was formed on the second to fourth toes of the right foot before surgery; **(b)** After debridement, the necrotic second to fourth toes of right foot was excised, and inserted antimicrobial bone cement; **(c)** Three weeks later, the bone cement was removed, and the wound was sutured directly, it was fresh, and the skin’s blood supply was adequate. **(d)** The wound of right foot had healed nicely after a month follow-up.

**Figure 10a** illustrates the case of a 74-year-old man with a 24-year history of DM who had diabetic gangrene in toes 2–4 of his right foot. The preoperative examination revealed that the WBC count, LPA level, and GGT level were within acceptable limits, and blood glucose was under control. Vascular surgery was performed to recanalize the patient’s right lower extremity, which also exhibited arterial embolism. The peripheral nerves of the damaged limbs did not experience any pain or numbness. After debridement, the necrotic 2–4 toes of the right foot were excised, and antimicrobial bone cement was inserted, as demonstrated in **Figure 10b**. Three weeks later, the bone cement was removed, and the wound was sutured directly. It was fresh, and the skin’s blood supply was adequate, as seen in **Figure 10c**. **Figure 10d** shows that the stitches were removed after 3 weeks, and the wound had healed adequately at the 1-month follow-up after surgery.

**Figure 11:**
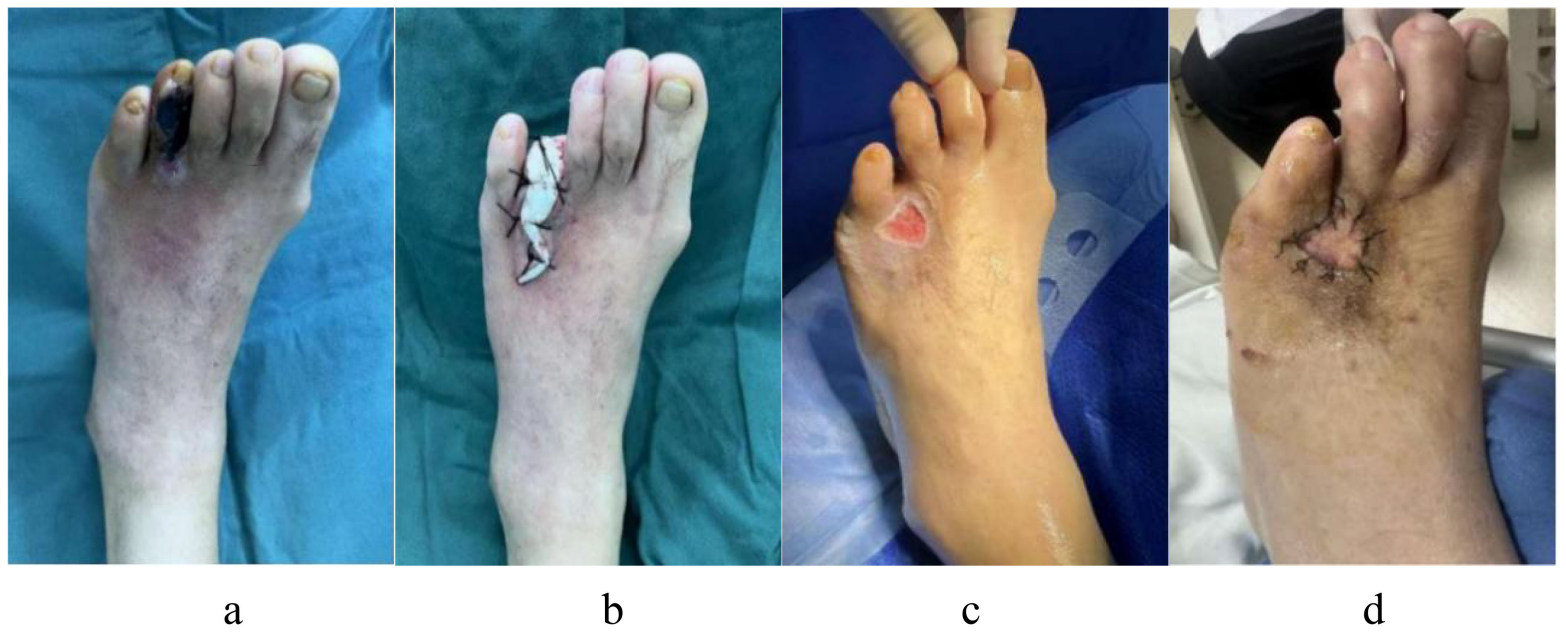
A 69-year-old man with a 20-year history of diabetes appeared diabetic gangrene in the fourth toe of his left foot, who accept first resection and antimicrobial bone cement operate, but a minor incision on the left foot did not heal. He had to receive another skin grafting surgery after the risk excluded. **(a)** Preoperative ulcerations of the fourth toe of the left foot; **(b)** After debridement, the necrotic fourth toes of left foot was excised, and inserted antimicrobial bone cement; **(c)** A minor incision on the left foot did not heal after operate. **(d)** The patient received recanalization of the blood vessels after evaluating the risk factors, then whose wound had healed nicely following skin grafting.

A 69-year-old man with a 20-year history of DM had diabetic gangrene in the fourth toe of his left foot, as depicted in **Figure 11a**. Preoperative assessment revealed that the WBC count was elevated, blood glucose levels were within normal limits, and LPA and GGT levels were also within normal limits. Recanalization was not performed before the amputation in this patient because the distal vascular disease in the left lower extremity was extensively embolized. Following anti-infective therapy, the WBC count normalized. A minor incision on the left foot, however, did not heal following the removal of the fourth toe and the application of antimicrobial bone cement, as seen in **Figure 11b, c**. Vascular surgery was performed to clear the blood vessels in the lower limbs after evaluating the risk factors. The blood supply to the damaged limbs had improved considerably, and the wound had healed sufficiently following skin grafting, as illustrated in **Figure 11d**.

### 4.2 Discussion

DFU is a severe complication of DM that considerably affects patients’ quality of life (31). Most patients require nontraumatic lower-extremity amputation owing to DFUs and subsequent infection, ultimately resulting in significant mortality (32, 33). A novel approach that has shown promising clinical outcomes is the use of antibacterial bone cement to treat DFUs (34). Bone cement, made of polymethyl methacrylate (PMMA), is a biomaterial that falls under the category of acrylic resin polymers. This material is prepared at room temperature by combining PMMA particle polymer with methyl methacrylate monomer, along with initiators, activators, and stabilizers (35). PMMA was initially used as a dental material in the 1930s and has since been increasingly applied in orthopedics (36). Nonetheless, because the bone cement lacks antibacterial qualities, it is vulnerable to infection and bacterial adherence at the bone–cement interface. Patients with DF are more prone to infections because of an imbalance in the body’s immunological response caused by blood vessel merging, neuropathy, and persistent hyperglycemia. Therefore, a specific amount of antimicrobial activity is provided for the treatment of DF wounds by adding antibacterial materials to the bone cement (37). To promote the healing of DFI wounds, the bone cement concurrently induces the surrounding tissue to develop a highly vascularized fibrous membrane that secretes various cytokines. These include bone morphogenetic protein 2, fibroblast growth factor, transforming growth factor β, and vascular endothelial cell growth factor. Furthermore, these cytokines encourage the production of type I collagen (38, 39).

Based on earlier studies on the risk factors for DF issues in patients with DM, several researchers have developed relevant prediction models. Their findings include risk factors for peripheral angiopathy of diabetes, diabetic peripheral neuropathy, high-density cholesterol, male sex, body mass index, glycosylated hemoglobin A1c, WBC, albumin, blood uric acid, fibrinogen, serum lactate dehydrogenase, ankle-brachial index, and C-reactive protein (11–13, 40–42). The risk factors identified by these researchers for patients with DM and difficult-to-treat DF, and the risk factors for amputation, are comparable to our screening and prediction of risk variables for the recurrence of DFUs treated with antibacterial bone cement. The risk factors identified by us are crucial for both surgical failure and the development of foot ulcers requiring amputation in patients with DM. Hence, managing these risk factors is essential to the success of bone–cement therapy for patients with DM in addition to preventing complications.

In our study, the WBC count was one of the risk indicators that may serve as a sensitive marker of acute inflammation, suggesting the onset and progression of inflammation. WBC levels are closely correlated with the degree of infection, and patients with DF exhibit higher WBC levels than those with simple DM. WBC is therefore one of the indices used to predict the probability of recurrence in patients with DM who had undergone antimicrobial bone-cement surgery (43, 44). Tasdighi’s study (45) found that LPA level was an independent risk factor for DF in patients with DM. The accumulation of cholesterol on peripheral artery walls, which causes atherosclerosis, and the overproduction of LPA may be linked to this discovery. These changes result in inadequate blood flow to the patient’s lower legs and feet, causing tissue damage and necrosis as well as extending their period of ischemia and hypoxia. One of the risk variables for postoperative recurrence investigated in this study is LPA. Furthermore, our findings revealed that GGT is a risk factor for postoperative recurrence. The Maeda study observed that elevated GGT may be associated with diabetic nephropathy (46). Moreover, Williams’ study found that the mortality rate for patients with DM surged when GTT levels increased (47). Pathological changes in the peripheral blood vessels and nerves of the lower limbs, which are responsible for the onset of DF and a significant determinant of its severity, are among the examined risk factors. Patients with DM exhibit distal nerve abnormalities in their lower extremities to varying degrees. According to several early-stage studies, peripheral neuropathy occurs in approximately 80% of individuals with DFUs (48). The last mechanism of cell destruction in diabetic neuropathy is thought to be oxidative stress. Chronic sensory neuropathy is the source of the marked decrease in foot pain. Consequently, the risk of foot injuries is considerably elevated (49, 50). Following antimicrobial bone-cement surgery, these patients are also at risk for recurrent foot injuries and wound recurrence. Atherosclerotic occlusive disease of the lower extremities is referred to as peripheral vascular disease and is common among individuals with DM. Peripheral vascular dysfunction plays a significant role in the development and progression of DF issues in individuals with DM (51). Patients with DM are more likely to have thicker capillary basement membranes, atherosclerosis, endothelial cell hyperplasia, and arteriolosclerosis. Therefore, a reduction in blood flow to the lower extremities may aggravate the disease and cause foot wounds to heal poorly (52). Following antimicrobial bone-cement surgery, these patients are susceptible to recurring infections and foot ulcers.

Despite its effectiveness, surgical treatment of DFUs using antibacterial bone cement is associated with a significant failure rate. However, a clinical prediction model has not yet been developed to select the most suitable individuals for treatment, eliminate risk factors for patients, prevent postoperative recurrence, and increase the surgical cure rate. In this study, the XGBoost model was developed and validated, demonstrating therapeutic potential in preventing postoperative recurrence and accurately assessing the risk of recurrence in patients with DF following antimicrobial bone-cement treatment.

Nevertheless, this study has several limitations. First, at this point, the study is a single-center investigation. If multicenter patient data can be added later, it will enhance the model’s predictive power by expanding the sample size and the range of variables investigated. Second, as this study is retrospective, it may be prone to systematic bias; future prospective research is required. Lastly, certain patients were eliminated from the research owing to missing data or loss to follow-up, which could have compromised the reliability of the statistical analyses.

## 5 Conclusion

Antibiotic bone-cement surgery has been proven to be an effective treatment method for DF. To improve the success rate of this operation, a clinical prediction model was developed, preoperative evaluations and interventions were conducted, and the risk of recurrence and reamputation was reduced. By collecting relevant data, peripheral vascular disease, peripheral neuropathy, GTT, LPA, and WBC count were screened, which were identified to be the risk factors. A comparative analysis revealed that XGBoost was the optimal prediction model for the preoperative evaluation and screening of patients, resulting in a clinically effective outcome. Overall, this study is one of the first or few cases in which the XGBoost method has been applied in this type of clinical research.

## Data Availability

The raw data supporting the conclusions of this article will be made available by the authors, without undue reservation.
